# Optimizing photovoltaic arrays: A tested dataset of newly manufactured PV modules for data-driven analysis and algorithm development

**DOI:** 10.1016/j.dib.2024.110482

**Published:** 2024-04-26

**Authors:** Ahmed Al Mansur, Md. Imamul Islam, Md. Sabbir Alam, Mohd Shawal Jadin, Zinat Sultana, M. M. Naushad Ali, ASM Shihavuddin

**Affiliations:** a Department of Electrical and Electronic Engineering, Green University of Bangladesh, Purbachal American City, Kanchon 1460, Bangladesh; b Faculty of Electrical Electronic Engineering, Universiti Malaysia Pahang Al-Sultan Abdullah, Pekan, 26600, Pahang, Malaysia; c Center for Research Innovation and Transformation(CRIT), Green University of Bangladesh, Purbachal American City, Kanchon 1460, Bangladesh

**Keywords:** New photovoltaic module, Electrical parameters of modules, Tested data, Commercial PV testing system, Mismatch power loss, Manufacturing tolerance

## Abstract

This data article presents a comprehensive dataset comprising experimentally tested characteristics of newly manufactured photovoltaic (PV) modules, which have been collected by using a commercial PV testing system from a solar panel manufacturer company. The PV testing system includes an artificial sunlight simulator to generate input light for the PV and the outputs of the PV are tested by a professional IV tracer in a darkroom environment maintaining IEC60904–9 standard. The dataset encompasses modules with power ratings of 10 W, 85 W, and 247 W, each represented by 40 individual module records. The tested and collected characteristics of each module include open circuit voltage, short circuit current, maximum power point voltage, maximum power point current, maximum power point power, and fill factor. The motivation for this dataset lies in addressing the challenges posed by manufacturing defects and *a* ± 5 % manufacturing tolerance, which can lead to mismatch power losses in newly installed PV arrays. These losses result in lower current in series strings and lower voltage in parallel branches, ultimately decreasing the array's output power. The dataset serves as a valuable resource for academic research, particularly in the domain of PV array optimization. To facilitate optimization efforts, different algorithms have been explored in the literature. This dataset supports the exploration of these optimization algorithms to find solutions that enhance the position of each module within the array, consequently increasing the overall output power and efficiency of the PV system. The objective is to mitigate mismatch power losses, which, if unaddressed, can contribute to increased degradation rates and early aging of PV modules. This dataset lays the groundwork for addressing critical PV array performance and efficiency issues. In future research, this dataset can be reused to explore and implement optimization algorithms, to improve the overall output power and lifespan of newly installed PV arrays. The smart solution proposed in [1], utilizing a genetic algorithm-based module arrangement, demonstrates promising results for maximizing PV array output power using this dataset.

Specifications Table

**Table:** 

Subject	Photovoltaic Systems, and Renewable Energy
Specific subject area	Solar Photovoltaic System
Type of data	Table, Image, Graph, Figure.
Data collection	The dataset of solar modules was collected from a commercial PV manufacturer company. Three datasets were collected, each including forty solar modules: 10 W, 85 W, and 247 W. Every panel was tested in the laboratory using a professional flash-light testing system by maintaining IEC60904–9. The electrical specifications for every PV module that has been tested, include maximum power point (MPP) power, MPP voltage, MPP current, open circuit voltage, and short circuit current.
Data source location	The Electro Solar Power Ltd, Ashulia, Savar, Dhaka, Bangladesh. Latitude and longitude: 23.902, 90.305.
Data accessibility	Repository name: Harvard Dataverse Data identification number: 10.7910/DVN/DJGXXZ Direct URL to data: https://dataverse.harvard.edu/dataset.xhtml?persistentId=doi:10.7910/DVN/DJGXXZ
Related research article	Mansur, Ahmed Al, Md Ruhul Amin, and Kazi Khairul Islam. “Performance comparison of mismatch power loss minimization techniques in series-parallel PV array configurations.” *Energies* 12, no. 5 (2019): 874. https://doi.org/10.3390/en12050874 [1]

## 1 Value of the Data

• The dataset provides a valuable look at the performance characteristics of newly manufactured photovoltaic modules in real-world scenarios. By using this data, researchers may improve the accuracy of PV array simulations by bridging the gap between theoretical models and real-world issues.
• These datasets of small (10 W), medium (85 W), and large (247 W) PV modules can serve as a baseline for algorithm development for researchers investigating optimization algorithms for photovoltaic panels. The wide range of power rates and exacting measurements offer a solid platform for optimizing PV array output power through testing and refinement of optimization techniques.
• The development of solutions to reduce mismatch power losses in recently installed PV arrays depends on these datasets. By understanding the performance variations within and across power rates, researchers can propose data-driven strategies to optimize module arrangement and improve overall PV array efficiency.

## 2 Background

The motivation behind compiling the dataset of newly manufactured photovoltaic (PV) modules has arisen from the imperative need to address the mismatched power losses in newly installed PV arrays. Various manufacturing defects and ±3 % manufacturing tolerance [2] for polycrystalline PV modules contribute to mismatch losses, resulting in lower current in series strings and lower voltage in parallel branches. Which ultimately diminishes the overall output power of the PV array. Moreover, the mismatched power loss [3] can increase the degradation rate of PV modules which will increase the aging rate of new modules, and as a result, the early aging of PV modules will be more than expected. However, to investigate and mitigate these issues, various optimization algorithms have been explored in the literature. The purpose of these optimization techniques [4] is to improve the positioning of each module in the array, thereby increasing the output power of the array and overall efficiency. Since the module rearrangement technique based on the optimization algorithm is a smart solution to reduce the early aging of the PV panels and the mismatch loss, hence these datasets are crucial for academic research as well as for commercial PV power plant applications. The scientifically published paper [1] demonstrates that the genetic algorithm-based module arrangement outperformed other conventional algorithms in maximizing the output power of the PV array. Thus, this data article illustrates the favorable outcomes that ensued from the findings and analysis of the article [1].

## 3 Data Description

The dataset is derived from experimentally tested newly manufactured PV modules obtained from Electro Solar Power Ltd (ESPL), Dhaka, Bangladesh. The datasets were collected using a solar sun simulator, ensuring adherence to ISO standards for reliable and consistent measurements. The dataset repository [5] contains three different power-rated solar PV modules such as small (10 W), medium (85 W), and large (247 W). The data file is an Excel file, which has three different sheets. The first, second, and third sheets contain the 247 W, 85 W, and 10 W power-rated modules test data respectively. Each data sheet contains forty individual PV module parameters. The characteristics of an individual PV module, including a) Open Circuit Voltage (Voc), b) Short Circuit Current (Isc), c) Maximum Power Point Voltage (Vmp), d) Maximum Power Point Current (Imp), e) Maximum power (Pm), and f) Fill Factor (FF). Each datasheet contains the same nameplate values for forty PV modules with the same ratings. However, the tested data shows some deviations in each parameter due to the manufacturing tolerances or faults. During the manufacturing process, all the PV cells inside a PV module are not the same in size which is considered as a manufacturing tolerance typically ± (3 to 5)%. This results in the variation in the output parameters ± (3 to 5)% of the modules even though the leveling of the rating are same by the manufacturer. These variations result in lower current in series strings and lower voltage in parallel branches, ultimately decreasing the array's output power. Which causes a significant amount of mismatch power losses in the large PV arrays. The average value of the module parameters of all three datasets 247 W, 85 W, and 10 W are tabulated in Table 1.

**Table 1:** The average value of electrical parameters of 247 W, 85 W, and 10 W modules.

Module rating (W)	The average value of the module parameters					
	Voc(V)	Isc(A)	Vmp(V)	Imp(A)	Pm(W)	FF (%)
10	10.42	1.29	8.61	1.18	10.19	75.5
85	21.38	5.35	16.99	5.06	86.03	75.14
247	40.53	7.5	34.09	7.24	247.1	80.81

**Fig. 1:**
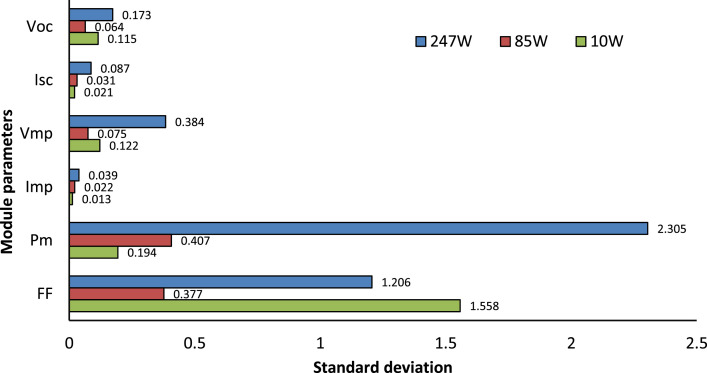
Standard deviation of different parameters of 247 W, 85 W and 10 W modules.

**Fig. 2:**
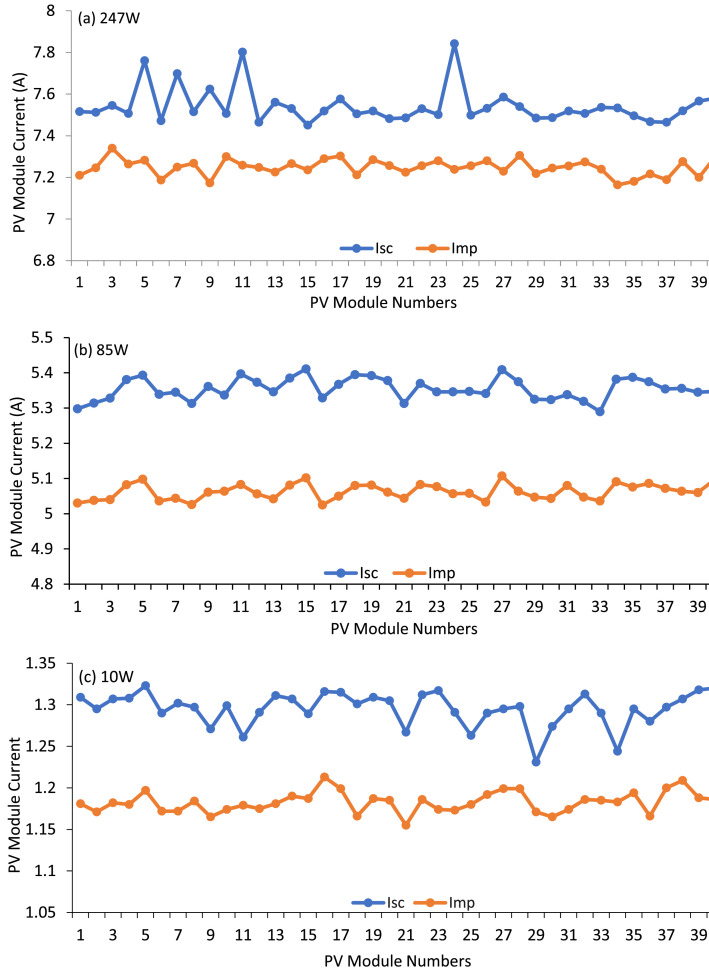
Short Circuit Current and Maximum Power Point Current of (a) 247 W, (b) 85 W, (c) 10 W PV modules.

**Fig. 3:**
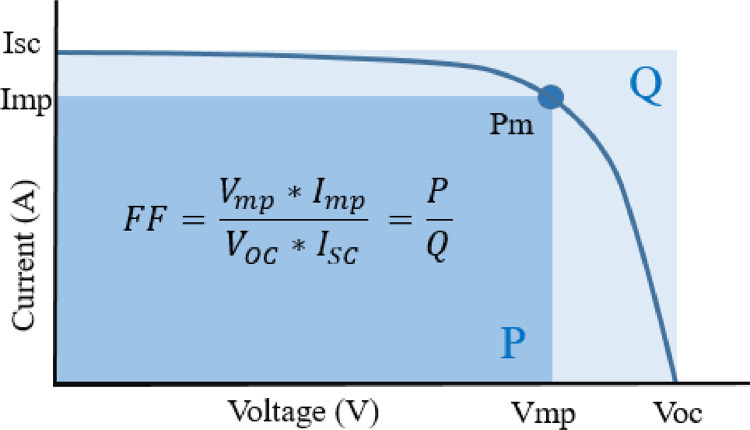
Fill factor of solar panel.

**Fig. 4:**
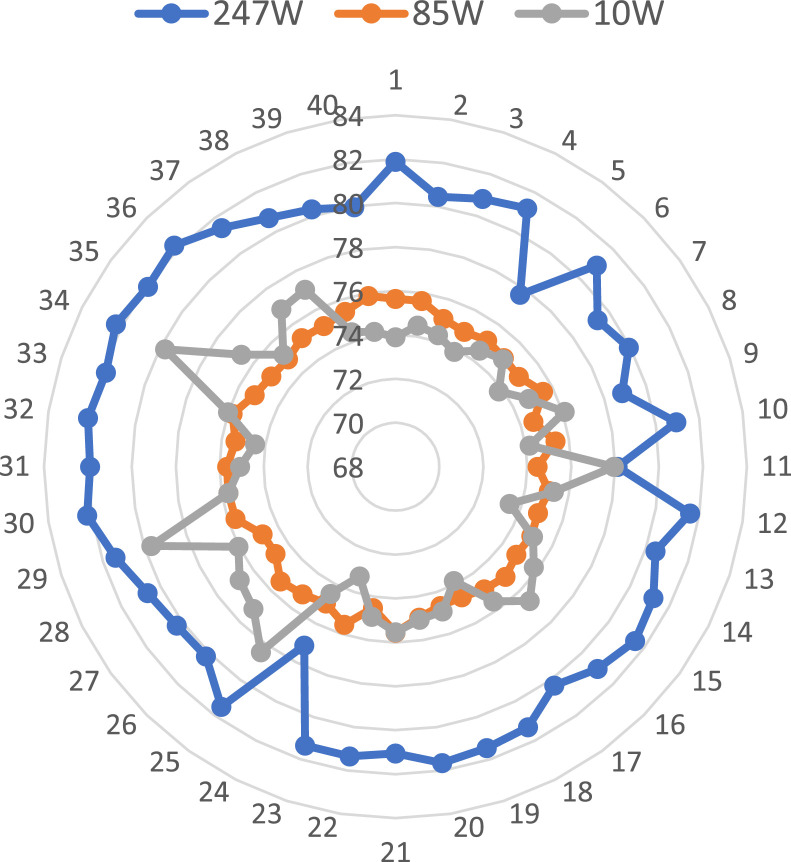
Comparison of fill factor of 247 W, 85 W and 10 W PV modules.

An interesting parameter of the datasets is standard deviation (SD) which is correlated with the power loss of the PV array. The standard deviation of all parameters such as Voc, Isc, Vmp, Imp, Pm, and FF for three different datasets of 247 W, 85 W, and 10 W modules have been plotted in Fig. 1. Among these six parameters, Imp and Isc are proportional to the module output power. Therefore, these two parameters (Imp and Isc) are plotted for the three datasets of 247 W, 85 W, and 10 W modules, which have been shown in Fig. 2. The FF of each PV module has been calculated using Equation 1. The relationship between the FF and the module parameters is shown in Fig. 3. A spider diagram has been illustrated in Fig. 4 to show the variation among the three different datasets of 247 W, 85 W, and 10 W PV modules. The selection of three different power-rated PV modules as small (10 W), medium (85 W), and large (247 W) PV modules are very significant for this type of research work. To identify the relation between mismatch power losses and power rating of the array modules. Besides a strong foundation for testing and improving optimization methods to maximize PV array output power is provided by the extensive range of power rates and precise measurements.

(1) $$FF=\frac{V_{mp}\;*\;I_{mp}}{V_{OC}\;*\;I_{SC}}\;=\frac{P}{Q}$$

## 4 Experimental Design, Materials and Methods

**Fig. 5:**
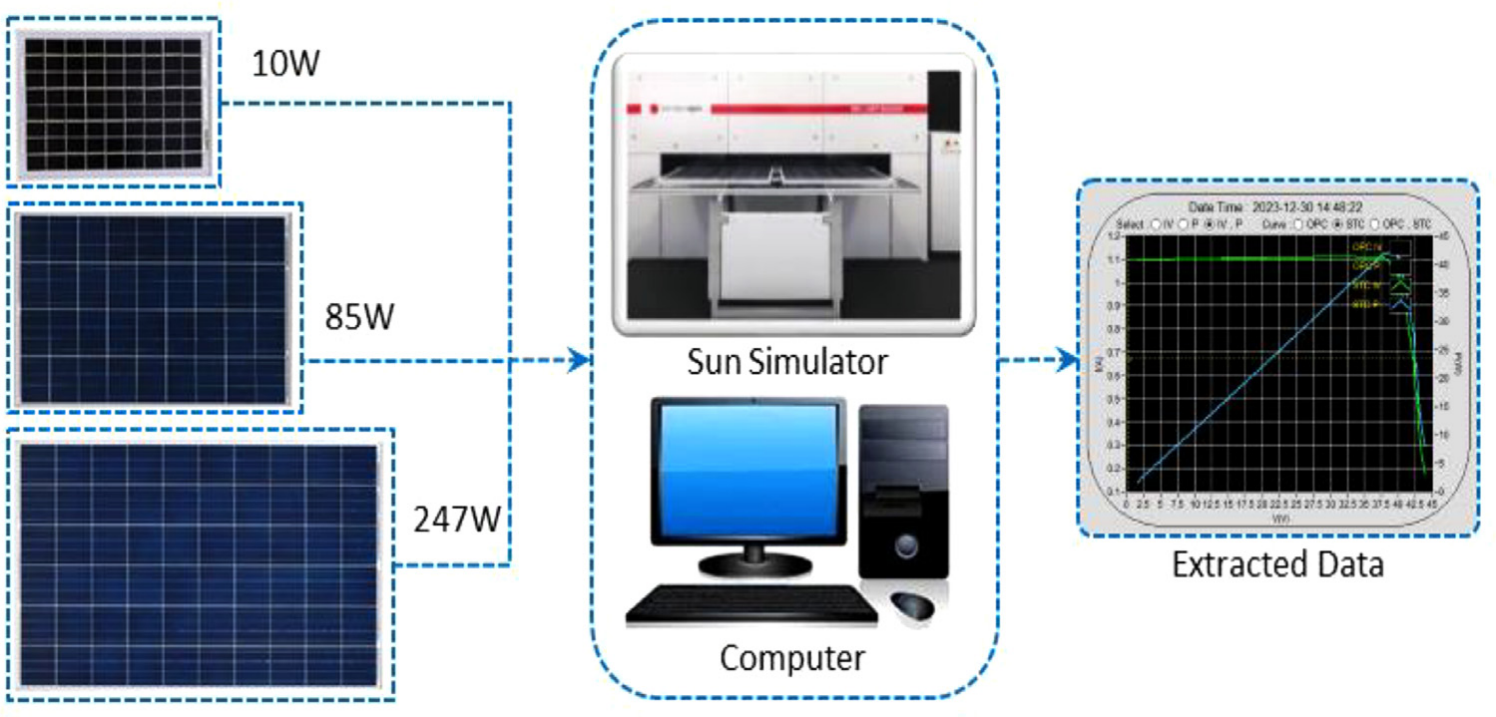
Experimental process of 10 W, 85 W and 247 W PV modules testing at solar panel manufacturer company.

The three newly acquired solar panels, having different power ratings of 10 W, 85 W, and 247 W, had been collected from a PV solar panel manufacturer, Electro Solar Power Limited (ESPL). According to the Standard Test Conditions (STC) [6], (Room temperature 25 °C, Irradiation 1000 W/m^2^, and Air Mass 1.5 G) the PV manufacturer company (Electro Group, Dhaka, Bangladesh) tested all the panels using a Xenon light-based artificial sun simulator. The block diagram of the experimental process has been deployed in Fig. 5. An I-V tracer was used for analyzing the output electrical parameter [7] of the different ratings of the photovoltaic modules and data was extracted and stored from the I-V tracer by a standard computer using the PV System Analyser software which has been provided by the manufacturing company. With Microsoft Excel, the collected data is accessible.

## Limitations

The data were collected from a single solar manufacturer (ESPL) in Dhaka, Bangladesh. The dataset encompasses three power rates (10 W, 85 W, and 247 W). Each PV module of the dataset provides the major specifications as Open Circuit Voltage (Voc), Short Circuit Current (Isc), Maximum Power Point Voltage (Vmp), Maximum Power Point Current (Imp), Maximum power (Pm), and Fill Factor (FF) but minor specifications like series resistance (Rs) and Parallel resistance (Rp) are not available.

## Ethics Statement

It has been confirmed by the authors, that all the datasets of this paper are tested experimentally considering the IEC standard by a PV manufacturer in Bangladesh. Moreover, the same data was used in the related published journal [1] by the corresponding author.

## CRediT authorship contribution statement

**Ahmed Al Mansur:** Conceptualization, Methodology, Writing – original draft, Data curation, Validation, Formal analysis, Investigation. **Md. Imamul Islam:** Data curation, Writing – review & editing. **Md. Sabbir Alam:** Conceptualization, Writing – original draft, Software, Formal analysis, Data curation. **Mohd Shawal Jadin:** Supervision, Conceptualization, Formal analysis. **Zinat Sultana:** Software, Formal analysis, Data curation. **M. M. Naushad Ali:** Formal analysis, Data curation. **ASM Shihavuddin:** Supervision, Project administration.

## Data Availability

Dataset of New Solar PV Modules_Harvard Dataverse (Original data) (Dataverse)
